# Constructing Distributed Hippocratic Video Databases for Privacy-Preserving Online Patient Training and Counseling

**DOI:** 10.1109/TITB.2009.2029695

**Published:** 2009-09-01

**Authors:** Jinye Peng, Noboru Babaguchi, Hangzai Luo, Yuli Gao, Jianping Fan

**Affiliations:** 1 School of Electronics and InformationNorthwestern Polytechnical University Xi'an 710072 China; 2 Department of Information and Communication TechnologiesGraduate School of EngineeringOsaka University Osaka 565-0871 Japan; 3 Software Engineering InstituteEast China Normal University Shanghai 200062 China; 4 Hewlett-Packard Laboratories Palo AltoCA 94304 USA; 5 Department of Computer ScienceUniversity of North Carolina Charlotte NC 28223 USA

**Keywords:** Online patient training and counseling, privacy-preserving classifier training and validation, privacy-preserving video database indexing, privacy-preserving video sharing

## Abstract

Digital video now plays an important role in supporting more profitable online patient training and counseling, and integration of patient training videos from multiple competitive organizations in the health care network will result in better offerings for patients. However, privacy concerns often prevent multiple competitive organizations from sharing and integrating their patient training videos. In addition, patients with infectious or chronic diseases may not want the online patient training organizations to identify who they are or even which video clips they are interested in. Thus, there is an urgent need to develop more effective techniques to protect both *video content privacy* and *access privacy*. In this paper, we have developed a new approach to construct a distributed Hippocratic video database system for supporting more profitable online patient training and counseling. First, a new database modeling approach is developed to support concept-oriented video database organization and assign a *degree of privacy* of the video content for each database level automatically. Second, a new algorithm is developed to protect the video content privacy at the level of individual video clip by filtering out the privacy-sensitive human objects automatically. In order to integrate the patient training videos from multiple competitive organizations for constructing a centralized video database indexing structure, a privacy-preserving video sharing scheme is developed to support privacy-preserving distributed classifier training and prevent the statistical inferences from the videos that are shared for cross-validation of video classifiers. Our experiments on large-scale video databases have also provided very convincing results.

## I. Introduction

To save the huge cost for national health care plan, online patient training and counseling are becoming very attractive by using digital videos to educate patients on early detection and self-treatment of their life-threatening diseases [1]. Because increasing the amounts of available patient training videos and increasing the diversity of video content may result in better offerings for patient training, it is very attractive to integrate the patient training videos from multiple competitive organizations in the health care network. However, privacy regulations, consumer backlash, and other privacy concerns often prevent multiple competitive organizations from sharing their patient training videos [1]–[10]. In addition, patients with infectious or chronic diseases, such as human immunodeficiency virus syndrome (AIDS), severe acute respiratory syndrome, bird flu, hepatitis, and diabetes, may not want the professional patient trainers and organizations to identify who they are or even which video clips they are interested in because disclosing private disease information may seriously affect their employment opportunities. Such privacy concerns may prevent the patients from using online training systems for early detection and self-treatment of their life-threatening infectious and chronic diseases. Thus, there is a strong need of new techniques that are capable of protecting both *access privacy* and *video content privacy*. Unfortunately, no comprehensive framework is available today to address the following inter-related issues effectively.

1) *Privacy-preserving video database indexing and retrieval:* To integrate the patient training videos from multiple competitive organizations for supporting more profitable online patient training and counseling, there is an urgent need of constructing a centralized indexing of the distributed video content [6]–[10], [11], [12]. As mentioned in [13], next-generation database systems referred to as *Hippocratic databases* should include responsibilities for both the data privacy and the access privacy.

2) *Privacy-preserving video sharing for distributed classifier training:* Classifying the video clips into a set of semantic video concepts is one promising approach to achieve concept-oriented video database indexing and access [9], [14], [15]. In order to learn the accurate concept models (i.e., video classifiers) for enabling a centralized indexing of the distributed video content, it is very important to support privacy-preserving video sharing among multiple competitive organizations.

**Fig. 1.**
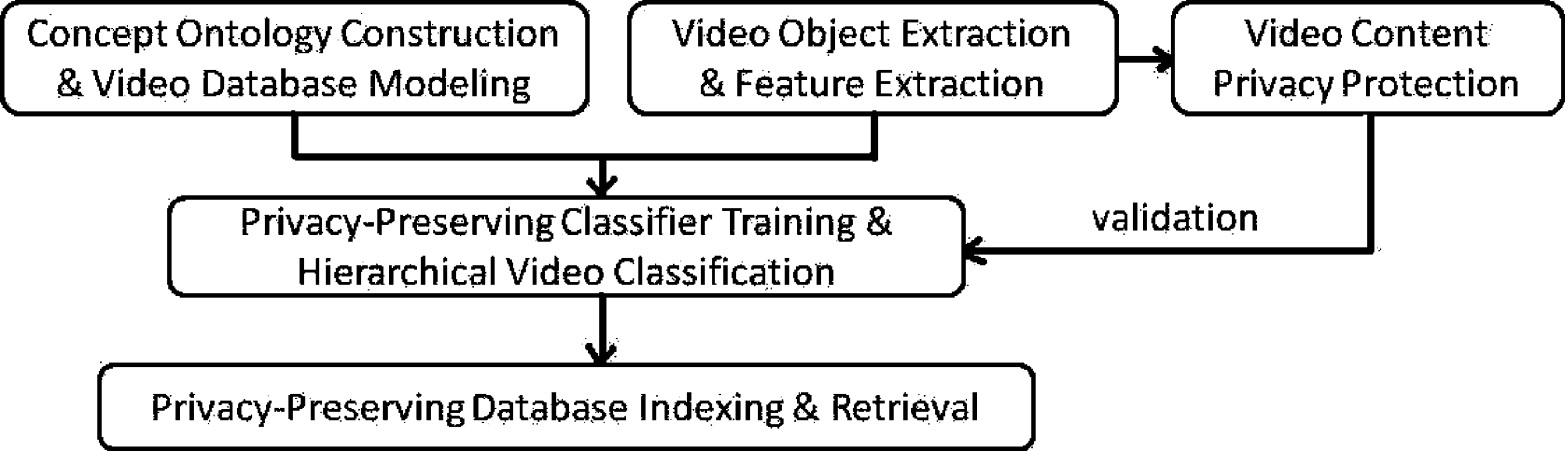
Flowchart of major components of our system.

Many techniques have been proposed recently to support privacy-preserving data sharing and mining [16]–[26]. By randomizing the original data or adopting secure multiparty computation, these techniques can protect the data privacy at the individual level and the algorithms are still able to recover aggregate information or to build data mining models. Data perturbation can protect individual data records, but it may also result in information loss as well as in privacy breaches due to the disclosure of perturbed data [16]–[20]. On the other hand, secure multiparty computation (SMC) approaches are too expensive to be useful for large-scale video database because of high communication costs [21]–[26]. Thus, these existing methods cannot directly be extended to enable privacy-preserving video sharing for distributed classifier training.

3) *Video content privacy protection:* The content privacy for the patient training videos consists of two major parts: a) privacy for the human objects who are shown in a video as the professional patient trainers or doctors; and b) privacy for the human objects who are shown in a video as the patients to illustrate the relevant clinic examples. When the patient training videos are released to the authorized patients or other parties, the video content privacy is disclosed [1], [2], [4]–[10].

To address these issues more effectively, we have developed a new system for supporting privacy-preserving online patient training and counseling and its major components are given in Fig. 1. This paper is organized as follows: Section II introduces our scheme for concept-oriented video database modeling by using concept ontology [14], [15]; Section III presents our privacy-preserving video sharing scheme to enable privacy-preserving distributed classifier training; Section IV introduces our framework on privacy-preserving centralized video database indexing and retrieval with a relaxed security model; Section V gives our experimental results; We conclude this paper at Section VI.

**Fig. 2.**
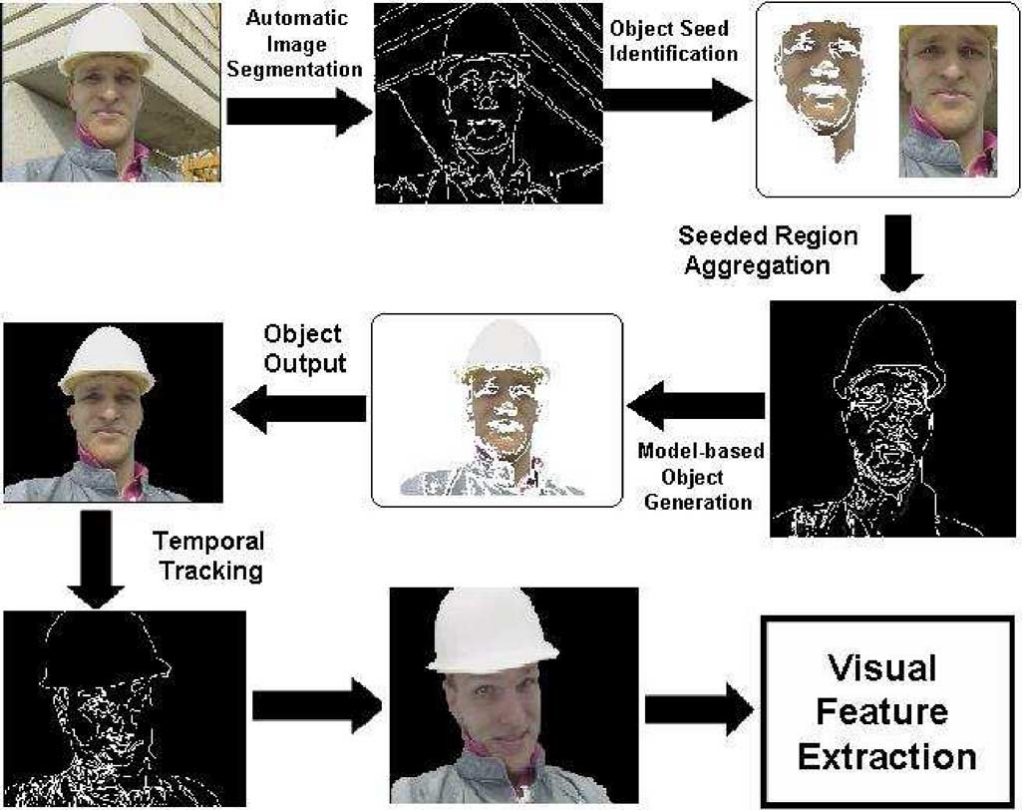
Flowchart of our algorithm for human object detection and tracking.

## II. Concept-Oriented Video Database Modeling

The video clips are first partitioned into a set of video shots automatically [14]. The video shots are used as the basic units for video content representation and feature extraction. To protect video content privacy, human objects are further extracted from the video clips automatically. The major steps of our human object detection function are illustrated in Fig. 2.

1) Automatic image segmentation is first performed on each video frame to obtain the homogeneous image regions [27].

2) The human face regions are then located by using traditional face detection techniques [28], [29]. The detected face regions are taken as the object seeds of human object determination [27].

3) The region relationship graph of object seeds (that is determined automatically in the image segmentation procedure) is then matched with the region constraint graph of human object (that is used for designing the human object generation function). If they are in good matching, the relevant image regions relevant are then aggregated to detect the human objects [27].

4) The detected human objects are then tracked among the video frames within the same video shot.

**Fig. 3.**
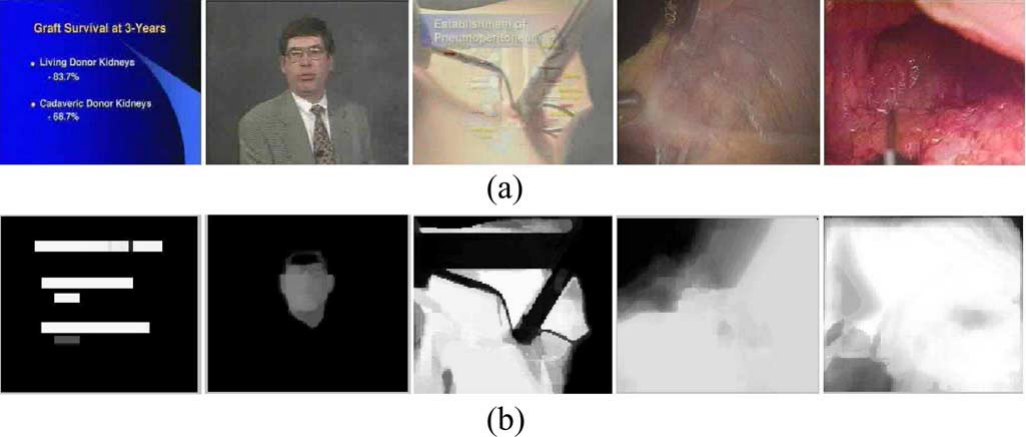
Confidence maps for object detection and tracking, where the white regions are used to indicate the detection confidences for the corresponding objects.

**Fig. 4.**
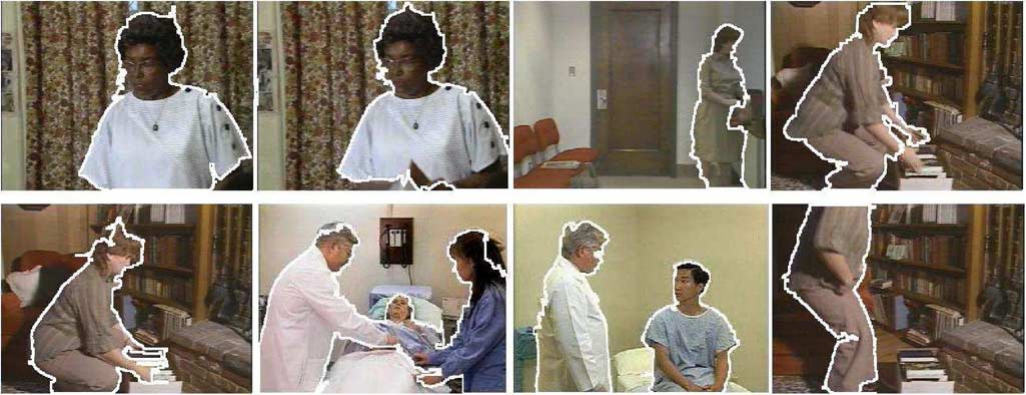
Experimental results for automatic human object detection.

Obviously, our human object detection function may fail in obtaining the meaningful human objects in some cases. To address this issue, two approaches can be used to improve human object detection: a) human–system interaction can be involved for defining the regions of human objects interactively [14]; and b) detection results for all the video frames within the same video shot are integrated and the relevant confidence maps for the detection results are calculated to provide a valuable information for human object detection as shown in Fig. 3. The confidence region is generated by transforming the relevant confidences for our detection results into a binary image via thresholding. Our experimental results on automatic human object detection and tracking are shown in Fig. 4. After the human objects are extracted, all the video clips are decomposed into a set of video shots with the associated human objects. It is well accepted that the visual properties of the videos are also important for video retrieval [9], [14], thus, both the global visual features and the local visual features are extracted for characterizing various visual properties of the semantic video concepts more precisely. In this paper, multiple feature subsets are extracted for video content representation: i) cumulative color histogram; ii) histogram of cumulative wavelet texture features; and iii) motion histogram.

A successful implementation of a *distributed Hippocratic video database system* for online patient training and counseling requires a well-defined database model for video indexing and privacy protection [1], [9]. Motivated by this observation, we have developed a concept-oriented framework for video database management that uses the notion of the *concept ontology* [14], [15]. The key idea of such concept-oriented video database organization is that the video clips are classified into a set of video concepts at different semantic levels [14], [15].

The concept ontology consists of two components: 1) semantic video concepts; and 2) their inter-concept contextual and logical relationships. The lower the level of a semantic video concept node, the narrower is its coverage of the subjects. The semantic video concepts at the first level of the concept ontology are named as *atomic video concepts*. Thus, the database nodes for the semantic video concepts at a lower semantic level can characterize more specific aspects of video content and they are easier to release the video content privacy and may have higher *degree of privacy*. To extract the text terms for semantic video concept interpretation, automatic entity extraction is first performed on large amounts of medical documents and medical experts are further involved to select the most meaningful video concepts interactively, so that these semantic video concepts are meaningful for domain experts and patient training.

The semantic similarity context $\varrho (C_i, C_j)$ between two semantic video concepts $C_i$ and $C_j$ is defined as

$$\varrho (C_i, C_j) = -P(C_i, C_j) \log {L(C_i, C_j)\over 2 D} \eqno{\hbox {(1)}}$$

where $L(C_i, C_j)$ is the length of the shortest path between the text terms for interpreting the semantic video concepts $C_i$ and $C_j$ in an one-direction IS-A taxonomy, $D$ is the maximum depth of such one-direction IS-A taxonomy [30], and $P(C_i, C_j)$ is the co-occurrence probability of the semantic video concepts $C_j$ and $C_i$ in the available medical documents. From this definition, one can observe that the semantic video concepts with smaller value of $L(\cdot, \cdot)$ on the taxonomy and higher co-occurrence probability $P(\cdot, \cdot)$ will correspond to stronger inter-concept semantic similarity context $\varrho (\cdot, \cdot)$.

Rather than using one single kernel function to characterize the diverse visual similarity contexts between the video clips, the visual similarity context between two video clips under each feature subset is characterized more precisely by using one particular basic kernel function. Finally, a mixture-of-kernels is used to integrate all these three basic kernel functions (i.e., three feature subsets) to characterize the diverse visual similarity contexts between the video clips more precisely

$$\kappa (x_h, x_k) = \sum_{i=1}^{3} \alpha_i \kappa_i(x_h, x_k) \eqno{\hbox {(2)}}$$

where $\alpha_i$ is the importance factor for the $i$th basic kernel function $\kappa_i(x_h, x_k)$.

For two semantic video concepts $C_i$ and $C_j$, their inter-concept visual similarity context $\gamma (C_i, C_j)$ can be determined by performing canonical correlation analysis [31] on their video sets $S_i$ and $S_j$:

$$\gamma (C_i, C_j) = \max_{\theta, \vartheta} {\theta^T \kappa (S_i) \kappa (S_j) \vartheta \over \sqrt{\theta^T\kappa^2(S_i)\theta \cdot \vartheta^T\kappa^2(S_j)\vartheta}} \eqno{\hbox {(3)}}$$

where $\theta$ and $\vartheta$ are the parameters for determining the optimal projection directions to maximize the correlations between two video sets $S_i$ and $S_j$ for $C_i$ and $C_j$, $\kappa (S_i)$ and $\kappa (S_j)$ are the cumulative kernel functions for characterizing the visual correlations between the videos in the same video sets $S_i$ and $S_j$

$$\kappa (S_i) = \sum_{x_l, x_m \in S_i} \kappa (x_l, x_m), \kappa (S_j) = \sum_{x_h, x_k \in S_j} \kappa (x_h, x_k) \eqno{\hbox {(4)}}$$

where the visual correlation between the video clips is defined as their kernel-based visual similarity.

The parameters $\theta$ and $\vartheta$ for determining the optimal projection directions are obtained automatically by solving the following eigenvalue equations:

$$\eqalignno{\kappa (S_i)\kappa (S_i) \theta - \lambda^2_{\theta } \kappa (S_i)\kappa (S_i) \theta &= 0 \cr \kappa (S_j)\kappa (S_j) \vartheta - \lambda^2_{\vartheta } \kappa (S_j)\kappa (S_j) \vartheta &= 0 &{\hbox{(5)}} }$$

where the eigenvalues $\lambda_{\theta }$ and $\lambda_{\vartheta }$ follow the additional constraint $\lambda_{\theta } = \lambda_{\vartheta }$.

**Fig. 5.**
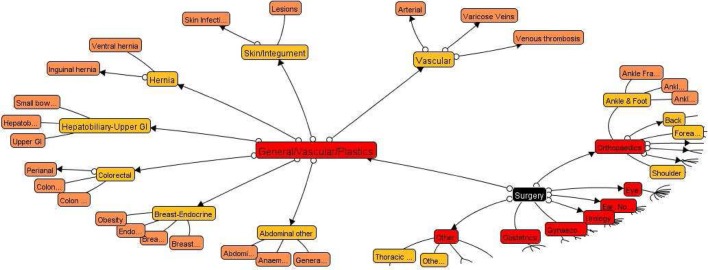
Parts of our concept ontology for organizing the patient training videos for hepatitis disease.

The inter-concept visual similarity context $\gamma (C_i, C_j)$ is first normalized into the same interval as the inter-concept semantic similarity context $\varrho (C_i, C_j)$. The inter-concept semantic similarity context and the inter-concept visual context are further integrated to achieve more precise characterization of the inter-concept similarity context $\varphi (C_i, C_j)$ between $C_i$ and $C_j$

$$\varphi (C_i, C_j) = \epsilon \varrho (C_i, C_j) + \eta \gamma (C_i, C_j), \epsilon + \eta = 1 \eqno{\hbox {(6)}}$$

where $\epsilon$ and $\eta$ are the relative importance factors for the inter-concept visual similarity context and the inter-concept semantic similarity context. One part of our domain-dependent concept ontology is given in Fig. 5.

The *advantages* of our approach for concept-oriented video database modeling and organization include: 1) by using the concept ontology for semantic video concept organization [14], [15], it is able to achieve concept-oriented video database indexing and retrieval; and 2) it is able to assign a *degree of privacy* of video content for each database level automatically. The lower-level video concepts on the concept ontology are used to describe more specific video content, thus, they are easier to release the privacy of video content and higher degrees of privacy should be assigned automatically.

## III. Privacy-Preserving Video Classifier Training

**Fig. 6.**
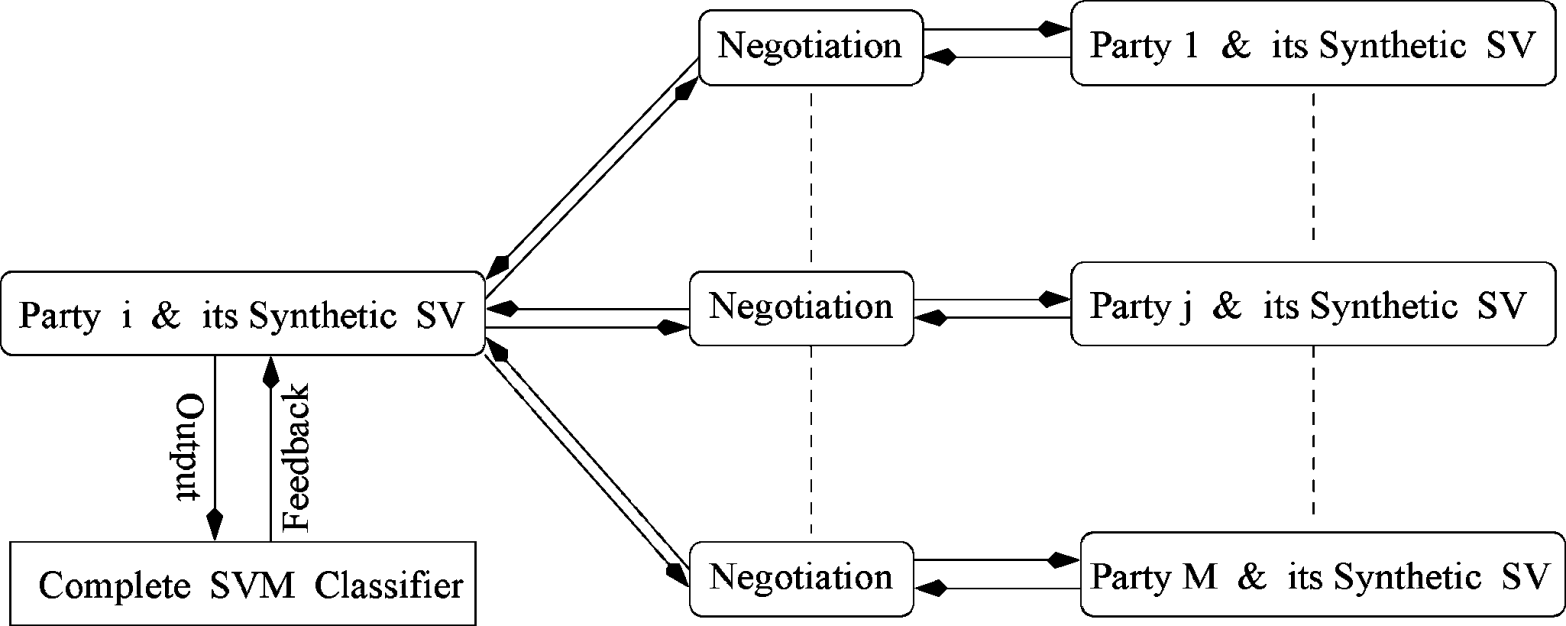
Our new approach for privacy-preserving distributed SVM classifier training, where SV represents support vectors.

To learn the classifiers collaboratively and assign the patient training video clips into the most relevant semantic video concepts automatically, we assume that there have $M$ collaboration parties in the health care network as shown in Fig. 6. By treating these $M$ collaboration parties as $M$ horizontally partitions of the available video sources, we have developed a new scheme to enable *privacy-preserving SVM classifier training*. Because the patient training videos (which are available at each of these $M$ collaboration parties in the collaboration network) are incomplete to characterize the diverse properties of the given semantic video concept precisely, it is impossible for any one of these $M$ collaboration parties to independently learn a *complete SVM classifier* with high prediction accuracy. Thus, it is very important for each party to integrate the most significant videos from other parties to validate and improve its own weak SVM classifier and learn a *complete SVM classifier* collaboratively. For a given classifier training task (i.e., learning the SVM classifier for one certain video concept of interest), our algorithm takes the following steps: 1) one weak SVM video classifier is learned locally at each party by using its own training videos; and 2) these $M$ weak SVM video classifiers are integrated as a strong classifier (i.e., complete SVM classifier) by sharing their support vectors (i.e., feature subsets that are extracted from the most significant video clips and are used to characterize the most significant and diversified properties of video content) for incremental SVM classifier training.

It is important to note that some common video clips may appear among these $M$ collaboration parties, thus sharing the original support vectors may allow the dishonest parties in the collaboration network to integrate their own video clips and the shared support vectors to inference the privacy of other parties. Thus there is an urgent need to support privacy-preserving sharing of the support vectors for learning the complete SVM classifier collaboratively.

For a given video concept $C_j$ on the concept ontology, *one-against-all* rule is used to label the training videos $\Omega_{c_{j}} = \{X_l, Y_l\vert l = 1, \ldots, N \}$ as positive videos or negative videos for learning the weak SVM classifier locally at each party. Each labeled video is a pair $(X_l, Y_l)$ that consists of three feature subsets $X_l$ and the semantic label $Y_l$. It is important to note that the confidential training videos (i.e., original labeled training videos) are not shared with other parties for collaborative classifier training.

For each party on the collaboration network, one weak SVM classifier is first learned by using its own labeled videos. For the positive videos $X_l$, that is, videos with $Y_l = +1$, transformation parameters $W$ and $b$ exist such that $f(X_l) = W \Phi (X_l) + b \ge + 1$. Similarly, for the negative videos $X_l$, that is, videos with $Y_l = - 1$, we have $f(X_l) = W \Phi (X_l) + b \le - 1$. $\Phi (X_l)$ is the function that maps $X_l$ into a higher dimensional space and the kernel function is defined as $\kappa (X_i, X_j) = \Phi (X_i)^T\Phi (X_j)$. In our current implementation, we select radial basis function (RBF), $\kappa (X_i, X_j) = \hbox{exp}(-\gamma \Vert X_i - X_j\Vert^2), \gamma > 0$. The margin between these two supporting planes is thus $2/\Vert W\Vert^2$. The weak SVM classifier is then designed for maximizing the margin with the constraints $f(X_l) = W \Phi (X_l) + b \ge + 1$ for the positive videos and $f(X_l) = W \Phi (X_l) + b \le - 1$ for the negative videos [32].

To incorporate the diversified videos from multiple parties for collaborative SVM classifier training, each party in the collaboration network has to share its support vectors with other parties. However, sharing the original support vectors with other parties is undesirable from privacy perspective, because the dishonest parties may leverage such *original support vectors* to inference the confidential information for other parties on the collaboration network, e.g., the dishonest parties may integrate such original support vectors and their own video clips to inference the privacy of other parties (i.e., whether other parties have similar video clips). There are two conflicting issues that must be addressed for achieving privacy-preserving SVM classifier training. On the one hand, sharing the original support vectors may leak confidential information of the original videos. On the other hand, the support vectors are used to interpret the underlying decision boundaries of the relevant week SVM classifier and characterize the principal properties of the original videos for the corresponding party, and thus sharing the original support vectors is critical for collaborative learning of the complete SVM classifier.

Based on this observation, we have developed a distributed framework to enable privacy-preserving SVM classifier training by generating and sharing *synthetic support vectors*. The synthetic support vectors are automatically generated from the original support vectors, and are used to approximate the decision boundaries (i.e., SVM margin boundaries) interpreted by the original support vectors [33]. Incorporating the synthetic support vectors for distributed SVM classifier training has at least two *advantages*: 1) using the synthetic values (i.e., the synthetic support vectors) to replace the original support vectors can effectively protect the video privacy because the shared synthetic support vectors are not extracted from the original videos; and 2) the synthetic support vectors can precisely preserve the underlying SVM decision boundaries that are interpreted by the original support vectors, and thus, they can be used to learn the complete SVM classifier.

The main problem for automatically generating such synthetic support vectors is to provide sufficient protection of the privacy for the individual value of each original support vector without damaging the important information (i.e., SVM margin boundaries) interpreted by the original support vectors. Based on this understanding, we have developed a novel framework to automatically generate the *synthetic support vectors* from the original support vectors by preserving the decision boundaries for the relevant weak SVM classifier. By replacing $W$ with $W =$ $\sum_{i=1}^N$ $\alpha_i$ $Y_i$ $\Phi (X_i)$ in the weak SVM classifier $f(X)= W \Phi (X) + b$, the dual form for the weak SVM classifier can be defined as

$$f(X) = \hbox{sgn} \left(\sum_{i=1}^N \alpha_i \kappa (X, X_i) + b\right) \eqno{\hbox {(7)}}$$

where $X_i$ is the $i$th video clip and $\kappa (X, X_i)$ is the underlying kernel function; the feature subsets for the video clips with $\alpha_i \ne 0$ are called the *support vectors*.

To generate the synthetic support vectors from the available weak SVM classifier $f(X)$, a *synthetic SVM classifier* $\psi (Z)$ is used to approximate $f(X)$ [i.e., approximate the decision boundaries of $f(X)$]

$$\psi (Z) = \hbox{sgn} \left(\sum_{i=1}^{N_z} \beta_i \kappa (Z, Z_i) + b\right) \eqno{\hbox {(8)}}$$

where $N_z < N$. To evaluate the approximation efficiency, two vector sets are defined as

$$\omega = \sum_{i=1}^N \alpha_i \Phi (X_i),\qquad \omega^{\prime } = \sum_{l=1}^{N_z} \beta_l \Phi (Z_l) \eqno{\hbox {(9)}}$$

where $\omega$ represents the set for the original support vectors, and $\omega^{\prime }$ represents the set of the synthetic support vectors that are used to approximate the underlying SVM decision boundaries interpreted by the set of the original support vectors $\omega$. Thus, the set of the synthetic support vectors $\omega^{\prime }$ can automatically be determined by minimizing

$$\eqalignno{\Vert \omega - \omega^{\prime }\Vert^2 &= \sum_{i,l=1}^N \alpha_i\alpha_l \kappa (X_i, X_l) + \sum_{i,l=1}^{N_z} \beta_i\beta_l \kappa (Z_i, Z_l)\cr &\quad- 2 \sum_{i=1}^N \sum_{l=1}^{N_z} \alpha_i\beta_l \kappa (X_i, Z_l) &{\hbox {(10)}} }$$

The crucial point is that even, if $\Phi (\cdot)$ is not given explicitly, (10) can be computed and minimized in terms of the kernel function [33].

In our current implementation, we have incorporated a novel iteration approach to generate the synthetic support vectors from the set of the original support vectors by minimizing the criterion function $\Vert \omega - \omega^{\prime }\Vert^2$. Our iteration framework takes the following major steps.

1) The first approximation, that is, $\beta \Phi (Z)$, $N_z = 1$, can be achieved by minimizing

$$\Vert \omega - \omega^{\prime }\Vert^2 = \Vert \omega - \beta \Phi (Z)\Vert^2. \eqno{\hbox {(11)}}$$

Rather than minimizing $\Vert \omega - \beta \Phi (Z)\Vert^2$, we can maximize the following new criterion function

$${(\omega \Phi (Z))^2\over (\Phi (Z) \Phi (Z))} \eqno{\hbox {(12)}}$$

Once the maximum of (12) is obtained, the value of $\beta$ is determined by

$$\beta = {(f(X) \psi (Z))\over (\psi (Z) \psi (Z))} . \eqno{\hbox {(13)}}$$

Because the RBF kernel is used in our current implementation, the relationship between the synthetic support vectors and the original support vectors can be obtained as

$$Z = {\sum_{i=1}^N \alpha_i \hbox{exp}(-\Vert Y_i - Z\Vert^2/(2\sigma^2))Y_i\over \sum_{i=1}^N \alpha_i \hbox{exp}(-\Vert Y_i - Z\Vert^2/(2\sigma^2))} \eqno{\hbox {(14)}}$$

and it devises an iteration relationship as

$$Z_{m+1} = {\sum_{i=1}^N \alpha_i \hbox{exp}(-\Vert Y_i - Z_m\Vert^2/(2\sigma^2))Y_i\over \sum_{i=1}^N \alpha_i \hbox{exp}(-\Vert Y_i - Z_m\Vert^2/(2\sigma^2))} . \eqno{\hbox {(15)}}$$

From above equation, one can observe that the value for each synthetic support vector is approximated by using a linear combination of all the original support vectors in $\omega$ and the synthetic support vectors that are previously used to approximate the underlying SVM decision boundaries. This linear combination process provides two significant benefits: a) it can suppress the privacy-sensitive values for the original support vectors and limit the privacy disclosure risk effectively; and b) it can enable a negotiable approach for incremental synthetic support vector generation, that is, generating and sharing different versions of synthetic support vectors with different levels of approximation efficiency (i.e., with different numbers of synthetic support vectors) for different parties or the same party at different negotiation loops according to their individual negotiation agreements.

2) Once the first approximation of $\omega$ is available, the iteration relationship for achieving more accurate approximation of the underlying SVM decision boundaries at the next level can be defined as

$$\omega_{m+1} = \sum_{i=1}^N \alpha_i \Phi (X_i) - \sum_{l=1}^m \beta_l \Phi (Z_l). \eqno{\hbox {(16)}}$$

In the Gaussian RBF case, it is worth noting that the SVM decision boundaries that are approximated by using the synthetic support vectors can never be as good as the real decision boundaries interpreted by the original support vectors, i.e., no approximation with $N_z < N$ will lead to $\omega_{m+1} = 0$. This observation also provides an effective solution for privacy protection by selecting the optimal number of the synthetic support vectors to achieve a good balance between the privacy disclosure risk, the generation and transmission cost, and the approximation efficiency.

Once the synthetic support vectors are available, the coefficient $\beta$ is updated as follows:

$$\beta = \Sigma^{-1} \Xi \alpha \eqno{\hbox {(17)}}$$

where $\Sigma$ and $\Xi$ are matrices, the components of which can be obtained by

$$\Sigma_{ij} = \Phi (Z_i) \Phi (Z_j),\quad \Xi_{ij} = \Phi (Z_i) \Phi (X_j). \eqno{\hbox {(18)}}$$

The above iteration procedure for generating the synthetic support vectors is terminated when one of the following criteria is meet: 1) a good balance (i.e., an optimal value of $N_z$) is reached between the privacy disclosure risk, the approximation efficiency, and the generation and transmission cost; and 2) $\omega_{m+1}$ falls below a specified threshold.

By performing the above iterations, we can automatically generate a set of synthetic support vectors $\omega^{\prime } = \sum_{i=1}^{N_z} \beta_i \Phi (Z_i)$ to approximate the underlying SVM decision boundaries that are interpreted by a set of original support vectors $\omega = \sum_{i=1}^N \alpha_i \Phi (X_i)$. These synthetic support vectors in $\omega^{\prime }$ are then shared with the collaboration parties according to their individual negotiation agreements. The *complete SVM classifier* can finally be achieved by incorporating the synthetic support vectors for incremental SVM classifier training.

**Fig. 7.**
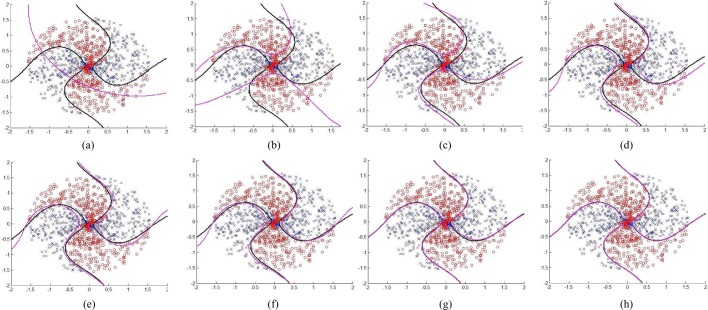
Using different numbers of synthetic support vectors for approximating the real SVM decision boundaries, where the red lines represent the SVM decision boundaries approximated by the synthetic support vectors and the black lines represent real SVM decision boundaries described by the original support vectors. (a) One synthetic support vector. (b) Six synthetic support vectors; (c) Ten synthetic support vectors. (d) Twelve synthetic support vectors. (e) Sixteen synthetic support vectors. (f) Twenty-one synthetic support vectors. (g) Thirty-two synthetic support vectors. (h) Thirty-six synthetic support vectors.

In order to achieve an acceptable tradeoff between the approximation accuracy (i.e., data utility and benefit for data sharing), the generation and transmission cost, and the privacy disclosure risk, we have developed a computational scheme to enable *automatic benefit/risk negotiation*. The outputs of our automatic benefit–risk negotiation scheme is further incorporated to control the procedures for synthetic support vector generation and sharing. Thus, each party in the collaboration network is able to generate and share different versions of the synthetic support vectors with different levels of approximation efficiency for different parties or the same party at different negotiation loops according to their individual negotiation agreements.

We have developed a computational approach to achieve a good balance between the approximation efficiency, the generation and transmission cost, and the privacy disclosure risk. The *approximation efficiency* (i.e., data utility and benefit for data sharing) is defined as

$$D(\omega, \omega^{\prime }) = \Vert \omega - \omega^{\prime }\Vert^2 = \left\Vert \sum_{i=1}^N \alpha_i \Phi (X_i) - \sum_{l=1}^{N_z} \beta_l \Phi (Z_l) \right\Vert^2 \eqno{\hbox {(19)}}$$

To illustrate the approximation efficiency, one synthetic dataset is used (i.e., using different numbers of synthetic support vectors to approximate the underlying SVM decision boundaries). This synthetic dataset consists of four spiral data groups found in pattern classification toolbox [34] and [35], and is generated by using the following steps: first, a set of synthetic data records are randomly generated from a uniform distribution of angles between 0 and $2\pi$ with radius between 0 and 1.5; second, the data records in the first and third quadrants are labeled as one class, and the rest data records are labeled as the other class; and finally, all these data records are transformed by linearly adding a radius-dependent angle to each data record, so that the added data records have larger angles than these original data records. As shown in Fig. 7, one can observe that the approximation efficiency can be improved step-wise by using more synthetic support vectors to approximate the underlying SVM margin boundaries interpreted by the original support vectors; this approximation procedure converges when an optimal number of synthetic support vectors is reached. By controlling the number of synthetic support vectors to be generated and to be shared with other parties, our negotiable framework for synthetic support vector generation and sharing is bi-directional, that is, each party has full control on its privacy disclosure risk and its individual benefit for data sharing.

From the experimental results shown in Fig. 7, one can observe that such approximation procedure converges to the underlying SVM decision boundaries interpreted by the original support vectors when the number of synthetic support vectors is close to 32, and the total number of original support vectors is 96. After this approximation procedure converges to the underlying SVM decision boundaries interpreted by the original support vectors, adding more synthetic support vectors does not result in significant improvements of the approximation efficiency. On the other hand, sharing more synthetic support vectors may also induce a higher cost for synthetic support vector generation and transmission. Thus the generation and transmission cost $\Upsilon (\omega, \omega^{\prime })$ is defined as CPU and $I$/$O$ cost for synthetic support vector generation and transmission. Obviously, $\Upsilon (\omega, \omega^{\prime })$ is a monotonically increasing function of the number of synthetic support vectors, i.e., $N_z$.

To quantify the privacy disclosure risk $\Re (\omega, \omega^{\prime })$, we have developed a computational approach to quantify three different types of privacy disclosure risk: 1) *re-identification disclosure risk* $\delta_r(\omega, \omega^{\prime })$: the risk of disclosing the one-to-one relationship between the synthetic support vectors and the original support vectors; 2) *linkage disclosure risk* $\rho (\omega, \omega^{\prime })$: the risk of re-identifying the values of the original support vectors by using the shared synthetic support vectors; and 3) *confidentiality-interval inference risk* $\phi (\omega, \omega^{\prime })$: the risk of disclosing the tight bounds of the interval values of the original support vectors

$$\eqalignno{\delta_r(\omega, \omega^{\prime }) &= \left\{\matrix{1,\quad X_i = Z_i\hfill\cr 0,\quad \hbox{otherwise}\hfill }\right. &{\hbox {(20)}}\cr \phi (\omega, \omega^{\prime }) &= \sum_{i=1}^N {1\over \log (1000\vert X_i -Z_i\vert)} &{\hbox {(21)}}\cr \rho (\omega, \omega^{\prime }) &= {1\over \log (1000\Delta (\omega, \omega^{\prime }))} &{\hbox {(22)}} }$$

where $X_i$ and $Z_i$ are the $i$th original support vector and the $i$th synthetic support vector, and $\Delta (\omega, \omega^{\prime })$ is the *information loss* incurred by the use of the synthetic support vectors to replace the original support vectors

$$\Delta (\omega, \omega^{\prime }) = {\sum_{i=1}^{N}\sum_{j=1}^{N_z} (X_i - Z_j)^2\over N N_z} . \eqno{\hbox {(23)}}$$

where $N$ and $N_z$ are the total numbers of the original support vectors and the synthetic support vectors. Thus the privacy disclosure risk $\Re (\omega, \omega)$ is defined as

$$\Re (\omega, \omega^{\prime }) = \alpha_1\delta_r(\omega, \omega^{\prime }) + \alpha_2\rho (\omega, \omega^{\prime }) + \alpha_3\phi (\omega, \omega^{\prime }) \eqno{\hbox {(24)}}$$

where $\alpha_1$, $\alpha_2$, and $\alpha_3$ are the weighting factors denoting the relative importance between $\delta_r(\omega, \omega^{\prime })$, $\rho (\omega, \omega^{\prime })$, and $\phi (\omega, \omega^{\prime })$, $\alpha_1 + \alpha_2 + \alpha_3 = 1$. To enable customizable privacy modeling, each party can select different values for these weighting factors according to their individual concerns of various privacy disclosure risks.

**Fig. 8.**
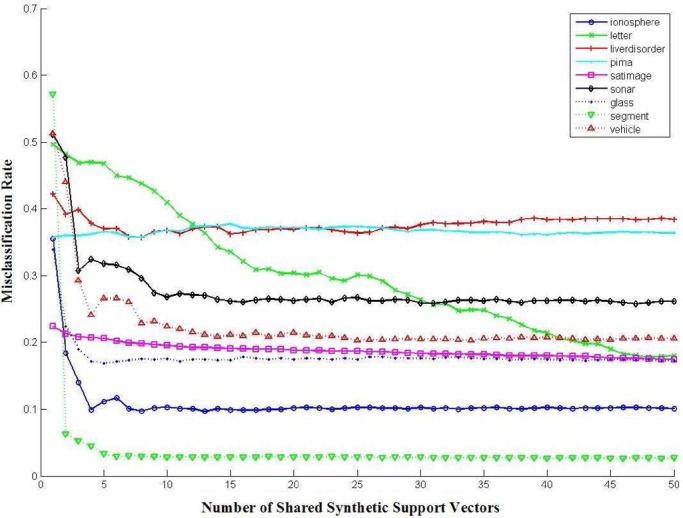
Empirical relationship between the misclassification rate $1 - \rho$ (i.e., classifier performance) and the number of synthetic support vectors to be shared.

Based on the above descriptions, it is very important to determine the optimal number $N_{\rm optimal}$ of the synthetic support vectors to be shared between two parties, and $N_{\rm optimal}$ is determined by achieving a good tradeoff between the privacy disclosure risk $\Re (\omega, \omega^{\prime })$, the generation and transmission cost $\Upsilon (\omega, \omega^{\prime })$, and the approximation efficiency $D(\omega, \omega^{\prime })$

$$\hbox{min} \{\Upsilon (\omega, \omega^{\prime }) + \lambda D(\omega, \omega^{\prime }) \}$$

subject to

$$\Re (\omega, \omega^{\prime }) < \delta \eqno{\hbox {(25)}}$$

where $\lambda$ is a weighting factor and $\delta$ is the upper bound of the privacy disclosure risk accepted by the corresponding party.

**Fig. 9.**
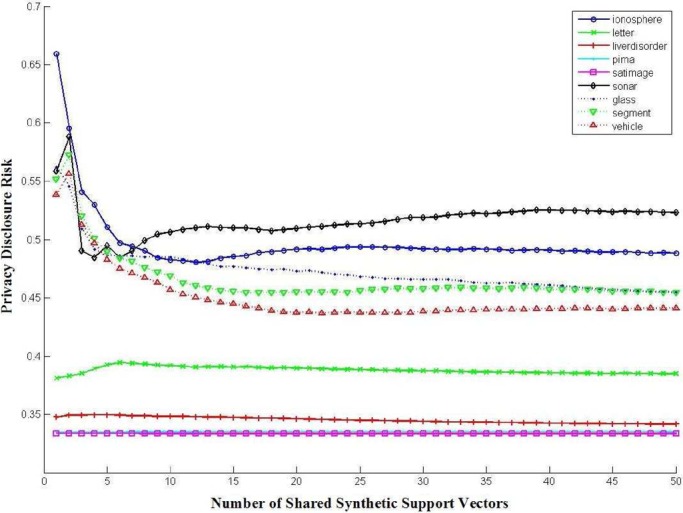
Empirical relationship between the privacy disclosure risk $\Re (\omega, \omega^{\prime })$ (i.e., classifier performance) and the number of synthetic support vectors to be shared.

Because different parties may obtain different versions of the synthetic support vectors with different levels of approximation efficiency from other parties in the collaboration network according to their individual negotiation agreements, they may finally learn different versions of the complete SVM classifier with different prediction powers according to their individual contributions for the given collaboration task, that is, a party by sharing more high-utility synthetic support vectors with other parties may also obtain more high-utility synthetic support vectors from other parties. Thus, each party in the collaboration network can have full control on its privacy disclosure risks and its individual benefits for data sharing. To illustrate the empirical relationship between the misclassification rate and the number of synthetic support vectors to be shared, we have tested our algorithms on multiple machine learning datasets and our results are given in Fig. 8. One can observe that sharing more synthetic support vectors can result in a complete SVM classifier with low-classification error rate. We have also obtained the empirical relationship between the privacy disclosure risk and the number of synthetic support vectors to be shared as shown in Fig. 9. One can observe that the privacy disclosure risk sequentially decreases with the number of synthetic support vectors which are shared for approximating the underlying SVM decision boundaries interpreted by the original support vectors. When more synthetic support vectors are shared to approximate the underlying SVM decision boundaries, it becomes more difficult to identify the one-to-one relationships between the original support vectors and the synthetic support vectors. However, the privacy disclosure risk consists of three individual parts: re-identification risk, linkage disclosure risk, and confidentiality-interval inference risk. Sharing more synthetic support vectors may also increase the ability to predict the value intervals of the original support vectors (i.e., confidentiality-interval inference risk) and induce higher linkage disclosure risk [36]–[39], and thus, we have also obtained a slight increasing of the privacy disclosure risk when more synthetic support vectors are shared as shown in Fig. 9.

**Fig. 10.**
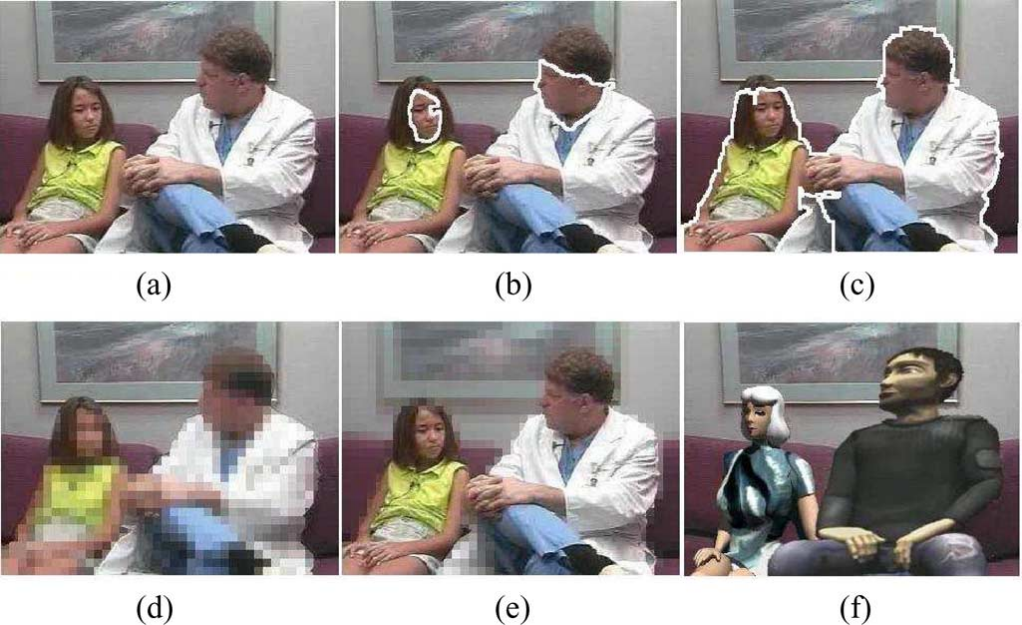
Experimental results for video content privacy protection. (a) Original video. (b) Face detection. (c) Human object detection. (d) Simple object blocking. (e) Simple background blocking. (f) Blurred video with virtual human objects.

**Fig. 11.**
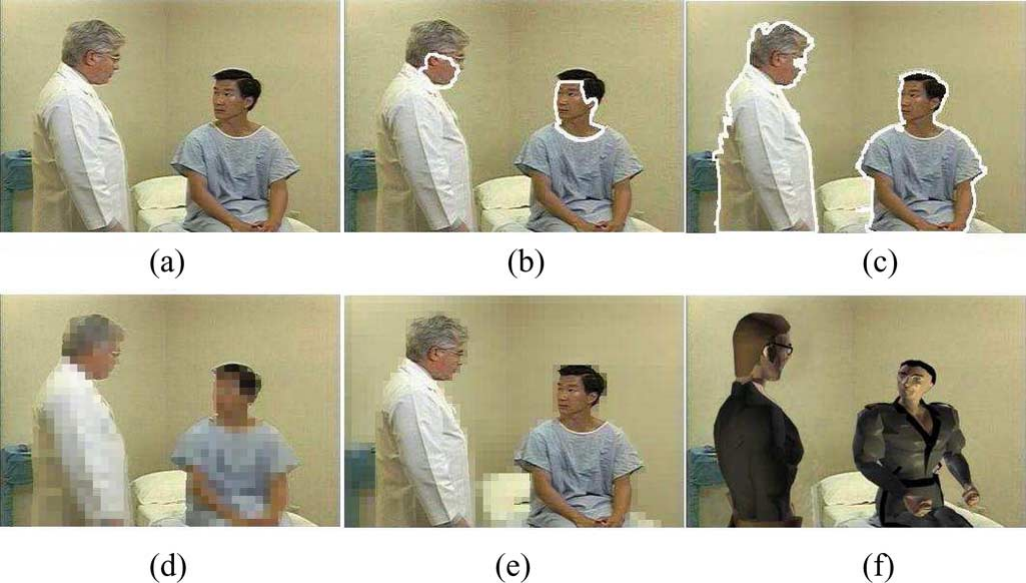
Experimental results for video content privacy protection. (a) Original video. (b) Face detection. (c) Human object detection. (d) Simple object blocking. (e) Simple background blocking. (f) Blurred video with virtual human objects.

**Fig. 12.**
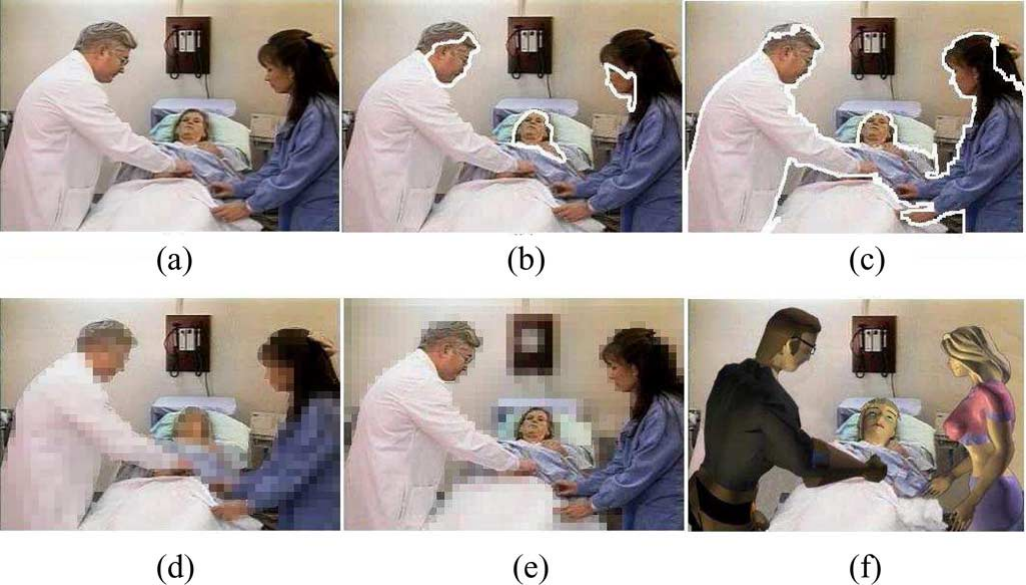
Experimental results for video content privacy protection. (a) Original video. (b) Face detection. (c) Human object detection. (d) Simple object blocking. (e) Simple background blocking. (f) Blurred video with virtual human objects.

**Fig. 13.**
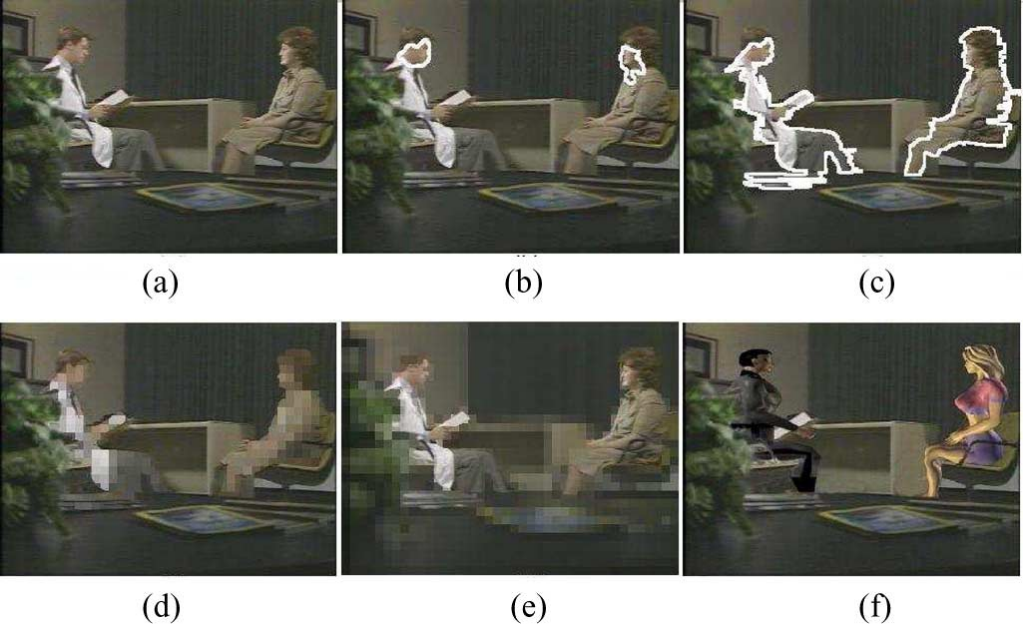
Experimental results for video content privacy protection. (a) Original video. (b) Face detection. (c) Human object detection. (d) Simple object blocking. (e) Simple background blocking. (f) Blurred video with virtual human objects.

To assess the effectiveness of the complete SVM classifier, it is very important for each party to share some video clips for cross-validation. After the human object detection function is available, it is then used to protect the content privacy at the individual video clip level. To filter out the privacy-sensitive human objects (i.e., doctors, professional patient trainers, patients in video), digital human models (i.e., virtual human objects) are used to replace the appearances of privacy-sensitive human objects in a video [1], [10]. Thus, the blurred video streams (i.e., video streams by using the virtual human objects to replace the appearances of privacy-sensitive human objects) are able to protect the privacy-sensitive information about who appear in the video. On the other hand, the blurred video streams are still able to provide necessary information about the real medical treatment procedure for one certain infectious disease and enable high-quality online patient training and counseling. Our experimental results on video content privacy protection are given in Figs. 10–13.

## IV. Privacy-Preserving Video Database Indexing and Retrieval

By using the underlying concept models (i.e., support vectors and SVM classifiers for video classification) for database node representation, our new framework for hierarchical video concept modeling and organization has provided an effective framework to enable concept-oriented video database indexing. After all the video clips are classified into the video concepts at different semantic levels, the concept ontology can further be incorporated to construct hierarchical video database indexing structure, where the nodes of semantic video concepts become the nodes of the video database at different semantic levels, upon which the root node of the video database can be constructed automatically.

The following techniques have been used to support *concept-oriented video database indexing*: 1) the support vectors for semantic video concept modeling are used to characterize the statistical property of the relevant database node (i.e., one certain video concept); 2) the concept ontology can be incorporated to determine the hierarchical structure for video database indexing (i.e., contextual relationships among the parent node and the children nodes); and 3) each database node is jointly described by multiple parameters such as keyword and support vectors.

To achieve a good balance between the access privacy and the access efficiency, we have developed a novel technique to assign the *degree of privacy* for each database level. The *degree of privacy* is defined as a monotonically increasing function of the sensitivity of video content, and thus the *degree of privacy* for the $l$th database level is defined as

$$S(l) = {{\left({l\over L} \right)}^{\alpha }\over {\left({l\over L} \right)}^{\alpha } + \left(1 - {l\over L} \right)^{\alpha}},\qquad \alpha > 1, l \in [0, L] \eqno{\hbox {(26)}}$$

where $L$ is the total number of database levels (i.e., depth of the database indexing structure from the root node to the leaf nodes), $\alpha$ is a constant to control the increasing rate of the degree of privacy, $S(0)$ = 0 when $l = 0$ (i.e., database root node cannot release any privacy information), and $S(L)$ = 1 when $l$ = $L$ (i.e., database leaf nodes may have the highest degree of privacy). From the database root node to the database leaf nodes, the degree of privacy may increase sequentially because the database nodes at a lower semantic level are used to characterize more specific aspects of video content with higher degree of privacy.

When the degree of privacy $S(l)$ for the $l$th database level is higher than a threshold $T_{\delta }$, $S(l)$ $\ge$ $T_{\delta }$, all the nodes at the $l$th database level are stored in a separate hash table, and the contextual relationship between the parent node and its children nodes is not indexed for the low-level database nodes with higher degree of privacy. For the high-level database nodes with low degree of privacy, it is very important to explicitly index the underlying contextual relationships between the parent node and its children nodes, so that higher access efficiency can be achieved for large-scale video database applications. Thus, the associated pointers can be used to index the higher-level database nodes when their degree of privacy $S(l)$ is less than the threshold $T_{\delta }$, $S(l)$ < $T_{\delta }$. Based on these observations, we have developed an effective technique to achieve a good balance between the access efficiency and the access privacy by selecting a suitable threshold $T_{\delta }$. In our current experiments, the threshold $T_{\delta }$ is set as $T_{\delta } = 0.35$ and good results have been obtained.

When the degree of privacy $S(l)$ for the $l$th database level is less than the threshold $T_{\delta }$, the contextual relationships between the parent node and its children nodes can be explicitly indexed and the following parameters are used to index the database node $Q$ at the $l$th database level

$$\left\{\matrix{\hbox{Node}\;\;\; \hbox{representation}\; 1: \left\{N_S, [(\hbox{Child}_i, Q_i)\vert i\in [1, N_s] \right\}\hfill \cr \cr \hbox{Child}_i:\;\; \hbox{keyword}\; L_i, \hbox{model}\; \hbox{parameters}\; \Theta_i\hfill } \right. \eqno{\hbox {(27)}}$$

where $Q_{i}$ is the pointer associated to the $i$th children node of $Q$, $N_S$ is the number of children nodes of $Q$, $[(\hbox{Child}_{1}$, $Q_1),$ …, $(\hbox{Child}_i$, $Q_i)$, …, $(\hbox{Child}_{N_s}$, $Q_{N_s})]$ is the entries for the children nodes of $Q$, $L_i$ is the keyword for interpreting the semantics of the $i$th child node of $Q$, $\Theta_i$ is the complete set of synthetic support vectors to characterize the statistical property and the decision boundaries for the $i$th child node of $Q$.

When the degree of privacy $S(k)$ for the $k$th database level is higher than the threshold $T_{\delta }$, all the nodes at the $k$th database level are stored in a separate hash table without the pointers associated to their parent nodes and children nodes

$$\left\{\matrix {\hbox{Node}\;\;\; \hbox{representation}\ 2: \left\{N_s, [\hbox{Node}_i\vert i \in [1, N_s]] \right\}\hfill \cr \cr \hbox{Node}_i:\;\; \hbox{keyword}\ L_i, \hbox{model} \hbox{parameters}\ \Theta_i\hfill } \right. \eqno{\hbox {(28)}}$$

where $\hbox{Node}_i$ indicates the $i$th node at the $(k+1)$th database level without indexing its contextual relationship with its parent node and children nodes.

After the privacy-preserving video database indexing structure is available, it is used to support more effective semi-private video retrieval over large-scale video databases. To prevent the participating video providers from tracking the patients' access interest, each video clip for online patient training is distributed on $M$ video servers (i.e, servers for $M$ participating video providers). To prevent the host video server from misusing the video clips shared from other competitive video providers, the video clips are encrypted by using cryptography techniques. To achieve a good balance between the access efficiency (i.e., it depends on the number of database nodes to be accessed) and the access privacy, we have proposed a relaxed security model to support low-cost semi-private video retrieval over large-scale video databases.

To reliably assure the access privacy, our distributed Hippocratic video database system is able to allow the patients to submit $\nu$ random queries to the centralized video database indexing structure. Instead of simply submitting one target query, the patients are allowed to submit $\nu -1$ randomly selected queries in addition to one target query. From the server's view, these $\nu$ random queries are independent, and the patients are able to reconstruct the target video clip from the query results obtained by these $\nu$ random queries. On the other hand, our distributed Hippocratic video database system is unable to identify which one of these $\nu$ random queries is the target query for the particular patient. In addition, these $\nu$ random queries are independently performed on the underlying centralized privacy-preserving video database indexing structure, and our Hippocratic video database can get the final result for each of these $\nu$ random queries from one of these $M$ video servers (i.e., participating video providers). Thus, each video server can observe only the query sent to it and the corresponding video server can get no information on the particular video clip that the patients are really interested in.

In order to prevent the video providers in the health care network from tracking the traversal path of a particular query, the access redundancy is introduced when the *degree of privacy* for the $l$th database level is higher than the predefined threshold $T_{\delta }$. Instead of simply retrieving one target node and its children nodes, our private video retrieval scheme can request the database server $\eta - 1$ randomly selected nodes in addition to the target node, all these $\eta$ database nodes are stored in the same hash table without the pointers associated to their parent nodes and children nodes. By introducing the access redundancy for access privacy protection, it is hard for the video server to identify which privacy-sensitive database node and particular video clips that the patients have accessed and finally got.

To achieve a good balance between the access redundancy (i.e., accessing more database nodes rather than only the target node and its children nodes) and the access privacy, we can define the *disclosure of access privacy* $\phi (\eta$, $\nu)$ as

$$\phi (\eta, \nu) = {1\over \nu \eta^{\Delta}} \eqno{\hbox {(29)}}$$

where $\Delta$ is the total number of database levels stored by using separate hash tables without using the pointers to identify their contextual relationships with their children nodes. When more database levels are stored by using separate hash tables (i.e., with large value of $\Delta$), the disclosure of access privacy $\phi (\eta$, $\nu)$ is reduced by introducing more access redundancy. In addition, submitting more random queries (i.e., with large value of $\nu$) is also able to reduce the disclosure of access privacy.

The access redundancy $\varphi (\eta$, $\nu)$ is defined as

$$\varphi (\eta, \nu) = c \nu \eta^{\Delta } \eqno{\hbox {(30)}}$$

where $c = 2.8$ is a predefined constant.

By quantifying the disclosure of access privacy and the access redundancy, the problem for enabling low-cost semi-private video retrieval reduces to choosing the appropriate values for $\eta$ and $\nu$, so that we can achieve a good balance between the access privacy and the access efficiency automatically. Our semi-private video retrieval technique may leak the patients' privacy when all these three conditions are reached simultaneously: 1) multiple queries for the same video concept are received from the same patient and these queries are recorded by the video providers; 2) these competitive video providers are collaborated (i.e., sharing the queries they received) to infer the patients' privacy; and 3) these competitive video providers know exactly who submitted the given query. When all these three conditions are reached simultaneously, these competitive video providers in the health care network can collaborate to infer the patients' query privacy. On the other hand, oblivious or history-independent database indexing structure can support private information retrieval [10]–[26], but it is too expensive to be useful for real applications. Thus, our future works will focus on developing more efficient oblivious database indexing structures which are able to protect the access privacy effectively while reducing the implementation cost significantly.

## V. Algorithm Evaluation

Our experimental *algorithm evaluation* focuses on: 1) evaluating the performance of our distributed framework for privacy-preserving classifier training; 2) evaluating the performance of our privacy-preserving video database indexing structure; and 3) evaluating the performance of our private video retrieval framework.

The *benchmark metric* for the classifier evaluation includes *precision* $\rho$ and *recall* $\varrho$. They are defined as

$$\varrho = {\vartheta \over \vartheta + \gamma },\quad \rho = {\vartheta \over \vartheta + \nu } \eqno{\hbox {(31)}}$$

where $\vartheta$ is the set of true positive samples that are related to the corresponding concept and are classified correctly, $\gamma$ is the set of true negative samples that are irrelevant to the corresponding concept and are classified incorrectly, and $\nu$ is the set of false positive samples that are related to the corresponding concept but are misclassified.

**Fig. 14.**
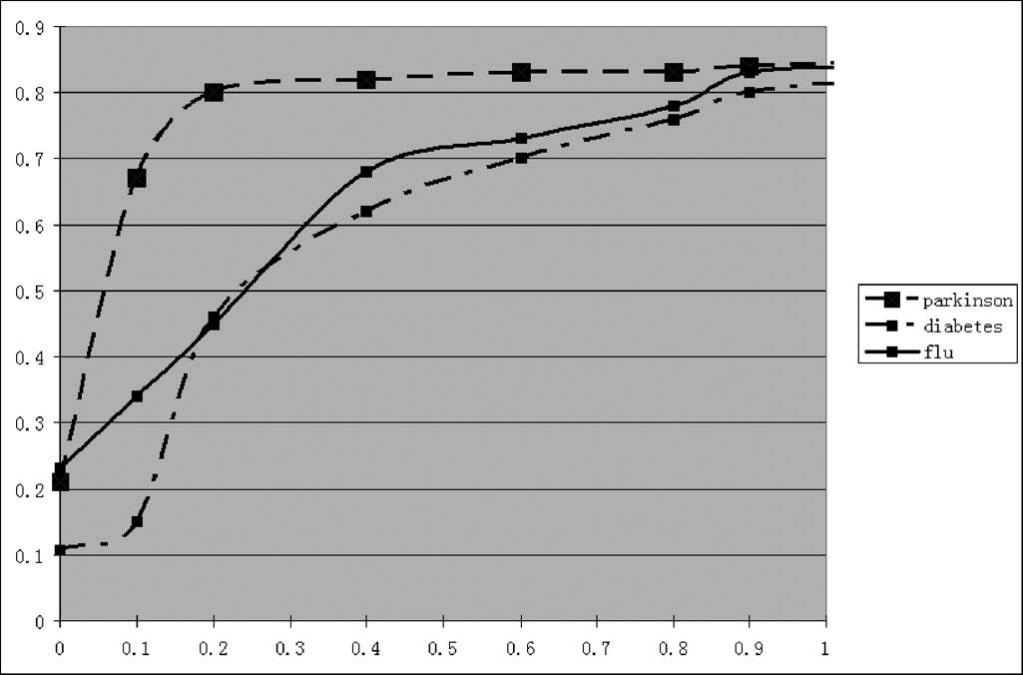
Empirical relationship between the classifier performance (i.e., precision $\rho$, $x$-axis) and the privacy disclosure ($y$-axis).

To evaluate our distributed framework for privacy preserving classifier training, the video set is partitioned into three individual groups and weak classifier training is first performed on these three individual data groups independently. The complete SVM classifier is then learned from the shared weak classifiers and the synthetic support vectors. One advantage of our distributed framework for privacy preserving classifier training is that it is able to control the privacy breaches by sharing different numbers of synthetic support vectors. Because these support vectors are used to approximate the decision boundaries of the complete SVM classifier for the given semantic video concept, sharing different number of support vectors can achieve different versions of the complete SVM classifier with different classification accuracy rates. Thus, a good balance between the disclosure of private information and the classification accuracy rate (i.e., depending on the quality of the complete SVM classifier) can be achieved effectively by sharing different resolutions of these weak classifiers. Based on this understanding, we have obtained the empirical relationships between the quality of the complete SVM classifier (i.e., precision of the complete SVM classifier) and the privacy disclosures as shown in Fig. 14. For three data sites, the total number of support vectors and the number of synthetic support vectors to be shared are given in Table I.

**Fig. 15.**
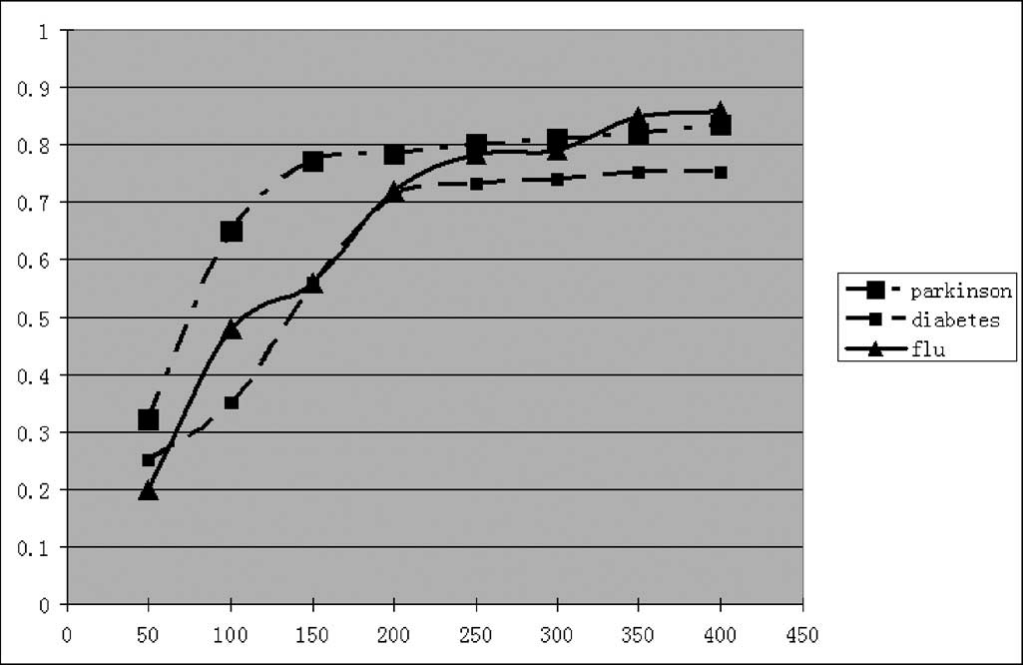
Empirical relationship between the privacy disclosure ($y$-axis) and the number of blurred test samples ($x$-axis) to be shared.

For validating the complete SVM classifier at the central site, each video provider has to share not only his/her weak classifier but also a limited number of blurred test videos. To prevent statistical inferences [36]–[39], we have also obtained the empirical relationships between the privacy disclosures and the number of blurred test videos to be shared as shown in Fig. 15. One can find that sharing more blurred test samples decreases the individual video provider's ability on controlling the statistical inferences and results in the privacy breaches [36]–[39].

**Table I:** Number of original support vectors $\phi$ and the number of support vectors to be shared $\varphi$

concepts	parkinson	diabetes	flu
(site 1: $\phi$	38	26	41
$\varphi$	31	21	36
(site 2) $\phi$	35	39	25
$\varphi$	29	32	18
(site 3) $\phi$	27	35	16
$\varphi$	23	28	12

**Fig. 16.**
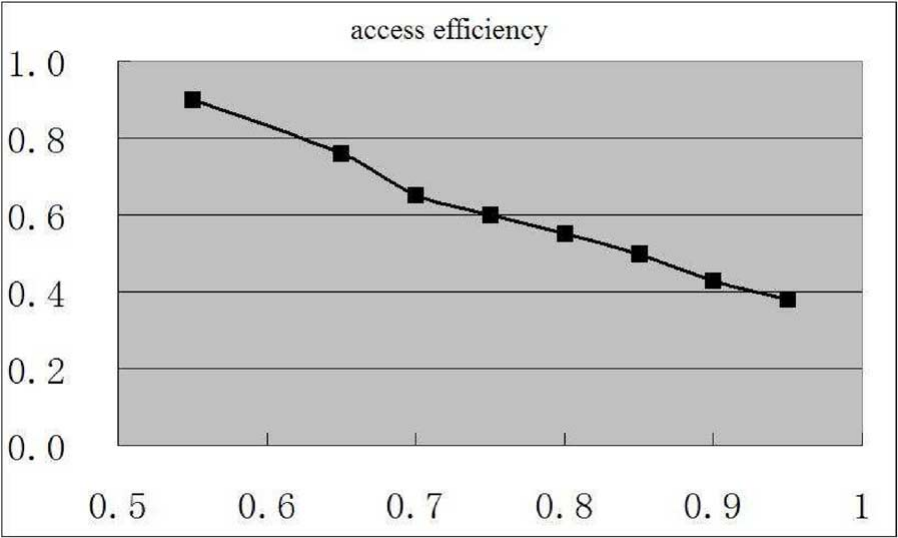
Empirical relationship between the access privacy ($y$-axis) and the access efficiency ($x$-axis) with $\nu = 5$, $\eta = 5$, and $\Delta = 3$.

To evaluate our framework for privacy-preserving video database indexing and private video retrieval, we have also tested our distributed Hippocratic video database system with 400 h medical education videos and our experimental results are given in Fig. 16. One can find that our system can achieve a good balance between the access privacy and the access efficiency by selecting the suitable values for $\nu$ and $\lambda$.

**Fig. 17.**
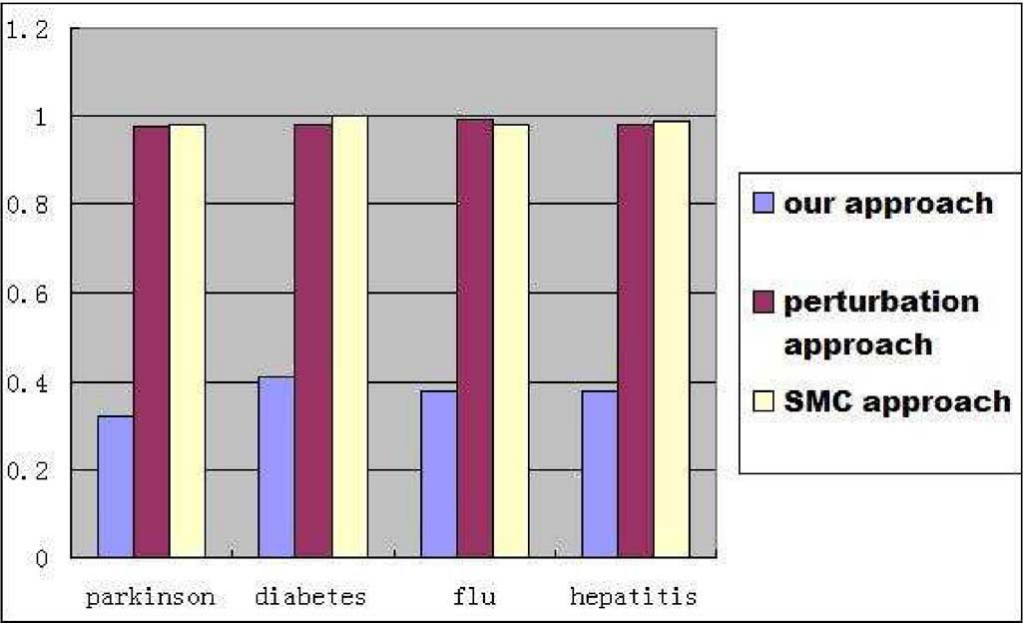
Comparison on transmission costs ($y$-axis) between our proposed framework and the perturbation and SMC approaches for distributed classifier training.

We have also compared the differences on the transmission costs between our proposed framework and the perturbation and SMC approaches. The transmission cost is defined as the percentage between the number of shared videos and the total number of videos that are needed for achieving accurate classifier training. As shown in Fig. 17, one can find that our proposed framework can reduce the transmission cost significantly because only a limited number of the blurred test videos are needed to be shared for classifier validation.

## VI. Conclusion

To enable privacy-preserving video sharing among multiple competitive video providers, we have developed a novel framework able to both protect the video content privacy and control the statistical inferences. By detecting the privacy-sensitive human objects automatically, our algorithm is able to effectively protect the video content privacy at the level of individual video clip. By determining the optimal size of blurred test videos for classifier validation, our proposed framework for privacy-preserving distributed classifier training is able to not only limit the privacy breaches but also improve the classifier's accuracy significantly. Our experiments in a specific domain of patient training videos have also provided very convincing results.

## Supplementary Material

Color versions of one or more of the figures in this paper are available online at http://ieeexplore.ieee.org.

## References

[1] Fan J., Luo H., Hacid M.-S., and Bertino E., “A novel approach for privacy-preserving video sharing,” in Proc. ACM CIKM, 2005, pp. 609–616.

[2] Newton E., Sweeney L., Malin B., “Preserving privacy by de identifying facial images,” School Comput. Sci., Carnegie Mellon Univ., Pittsburgh, PA, in Tech. Rep. CMU-CS-03-119, 2003.

[3] Newton E., Sweeney L., and Malin B., “Preserving privacy by de identifying facial images,” in IEEE Trans. Knowl. Data Eng., vol. 17, no. 2, pp. 232–243, Feb. 2005.

[4] Wickramasuriya J., Datt M., Mehrotra S., and Venkatasubramanian N., “Privacy protecting data collection in media spaces,” in Proc. ACM Multimedia, 2004, pp. 48–55.

[5] Senior A., Pankanti S., Hampapur A., Brown L., Tian Y., Ekin A., Blinkering surveillance: Enabling video privacy through computer vision, in IBM TR W0308-109, 2003.

[6] Boyle M., Edwards C., and Greenberg S., “The effects of filtered video on awareness and privacy,” in Proc. ACM Conf. Comput. Supported Cooperative Work, 2000, pp. 1–10.

[7] Adams A., “Users' perception of privacy in multimedia communication,” in Proc. ACM Conf. Comput.-Human Interaction, 1999, pp. 49–64.

[8] Patil S., Kobsa A., “The challenges in preserving privacy in awareness systems,” Univ. Calif., Davis, CA, in Tech. Rep. CSD-TR 92-010, 2003.

[9] Ebadollahi S., Chang S.-F., and Wu H., “Echocardiogram videos: Summarization, temporal segmentation and browsing,” in Proc. Int'l Conf. Image Process. (ICIP), 2002, pp. 1000–1103.

[10] Yu X., Chinomi K., Koshimizu T., Nitta N., Ito Y., Babaguchi N., “Privacy protecting visual processing for secure video surveillance,” presented at the ICIP, San Diego, CA, 2008.

[11] Agrawal R. and Srikant R., “Privacy-preserving data mining,” in Proc. ACM SIGMOD, 2000, pp. 439–450.

[12] Agrawal D. and Aggarwal C., “On the design and quantification of privacy preserving data mining algorithms,” in Proc. ACM PODS, 2001, pp. 249–255.

[13] Agrawal R. and Kiernan J., Srikant R., Xu Y., “Hippocratic databases,” in Proc. VLDB, 2002, pp. 281–292.

[14] Fan J., Elmagarmid A. K., Zhu X., Aref W. G., and Wu L., “ClassView: Hierarchical video shot classification, indexing and accessing,” in IEEE Trans. Multimedia, vol. 6, no. 1, pp. 70–86, Feb. 2004.

[15] Naphade M., Smith J. R., Tesic J., Chang S.-F., Hsu W., Kennedy L., Hauptmann A., and Curtis J., “Large-scale concept ontology for multimedia,” in IEEE Multimedia, vol. 13, no. 3, pp. 86–91, Jul-Sep 2006.

[16] Bawa M., Bayardo R. J., and Agrawal R., “Privacy-preserving indexing of documents on the networks,” in Proc. VLDB, 2003, pp. 922–933.

[17] Liew C. K., Choi U., and Liew C. J., “A data distortion by probability distribution,” in ACM Trans. Database Syst., vol. 10, pp. 395–411, 1985.

[18] Muralidhar K. and Sarathy R., “Security of random data perturbation methods,” in ACM Trans. Database Syst., vol. 24, no. 4, pp. 487–493, 1999.

[19] Arrawal R., Evfimievski A., and Srikant R., “Information sharing across private database,” in Proc. ACM SIGMOD, 2003, pp. 86–97.

[20] Merugu S. and Ghosh J., “Privacy-preserving distributed clustering using generative models,” in Proc. IEEE ICDM, 2003, pp. 218–230.

[21] Chor B., Goldreich O., Kushilevitz E., and Sudan M., “Protecting data privacy in private information retrieval schemes,” in Proc. ACM Symp. Theory Comput. (STOC), 1998, pp. 151–160.

[22] Naor M. and Teague V., “Anti-persistence: History independent data structures,” in Proc. ACM Symp. Theory Comput. (STOC), 2001, pp. 492–501.

[23] Micciancio D., “Oblivious data structure: Applications to cryptography,” in Proc. ACM Symp. Theory Comput. (STOC), 1997, pp. 456–464.

[24] Yao A., “How to generate and exchange secrets,” in Proc. IEEE Symp. Found. Comput. Sci. (FOCS), 1986, pp. 162–167.

[25] Goldreich O., Micali S., and Wigderson A., “How to play any mental game- a completeness theorem for protocols with honest majority,” in Proc. ACM Symp. Theory Comput. (STOC), 1987, pp. 218–229.

[26] Lindell Y. and Pinkas B., “Privacy preserving data mining,” in Proc. Annu. Int. Cryptology Conf. (CRYPTO), 2000, pp. 36–54.

[27] Fan J., Yau D. K. Y., Elmagarmid A. K., and Aref W. G., “Automatic image segmentation by integrating color edge detection and seeded region growing,” in IEEE Trans. Image Process., vol. 10, no. 10, pp. 1454–1466, Oct. 2001. 18255490 10.1109/83.951532

[28] Heisele B., Serre T., Prentice S., and Poggio T., “Hierarchical classification and feature reduction for fast face detection with SVM,” in Pattern Recog., vol. 36, pp. 2007–2017, 2003.

[29] Viola P. and Jones M., “Robust real-time face detection,” in Int. J. Comput. Vis., vol. 57, no. 2, pp. 137–154, 2004.

[30] Fellbaum C., WordNet: An Electronic Lexical Database, Boston, MA: MIT Press, 1998.

[31] Hardoon D. R., Szedmak S., Taylor J. Shawe, “Canonical correlation analysis: An overview with application to learning methods,” Univ. London, London, U.K., in Tech. Rep. CSD-TR-03-02, 2003. 10.1162/0899766042321814 15516276

[32] Platt J. C. “Probabilistic outputs for support vector machines comparisons to regularized likelihood methods” in Adavances in Large Margin Classifiers, Boston, MA: MIT Press, 1999.

[33] Scholkopf B., Knirsch P., Smola A., and Burges C. J. C., “Fast approximation of support vector kernel expansions, and an interpretation of clustering as approximation in feature spaces,” in Proc. DAGM Symp., Springer Lecture Notes Comput. Sci., 1998, pp. 125–132.

[34] Ripley B. D., “Neural network and related methods for classification,” in J. Royal Statistical Soc., Series B, vol. 56, pp. 409–456, 1994.

[35] Stork D. and Yom-Tov E., Computer Manual in MATLAB to Accompany Pattern Classification, New York: Wiley, 2004.

[36] Lindley D. V., “The choice of sample size,” in Statistician, vol. 46, no. 2, pp. 129–138, 1997.

[37] Adcock C. J., “Sample size determination: A review,” in Statistician, vol. 46, no. 2, pp. 261–283, 1997.

[38] Weiss R., “Bayesian sample size calculations for hypothesis testing,” in Statistician, vol. 46, no. 2, pp. 185–191, 1997.

[39] Guyon I., Makhoul J., Schwarts R., and Vapnik V., “What size test set gives good error rate estimates,” in IEEE Trans. Pattern Anal. Mach. Intell., vol. 20, no. 1, pp. 52–64, Jan. 1998.

